# Opportunistic vs selective feeding strategies of zooplankton under changing environmental conditions

**DOI:** 10.1093/plankt/fbad007

**Published:** 2023-02-22

**Authors:** Baptiste Serandour, Kinlan M G Jan, Andreas Novotny, Monika Winder

**Affiliations:** Department of Ecology, Environment and Plant Sciences, Stockholm University, Universitetsvägen 10A, SE-106 91, Stockholm, Sweden; Department of Ecology, Environment and Plant Sciences, Stockholm University, Universitetsvägen 10A, SE-106 91, Stockholm, Sweden; Department of Ecology, Environment and Plant Sciences, Stockholm University, Universitetsvägen 10A, SE-106 91, Stockholm, Sweden; Department of Ecology, Environment and Plant Sciences, Stockholm University, Universitetsvägen 10A, SE-106 91, Stockholm, Sweden

**Keywords:** zooplankton, metabarcoding, food-web, trophic niche, environmental gradient

## Abstract

The plankton community consists of diverse interacting species. The estimation of species interactions in nature is challenging. There is limited knowledge on how plankton interactions are influenced by environmental conditions because of limited understanding of zooplankton feeding strategies and factors affecting trophic interactions. In this study, we used DNA-metabarcoding to investigate trophic interactions in mesozooplankton predators and the influence of prey availability on their feeding behavior. We found that mesozooplankton feeding strategies vary within species across an environmental gradient. Some species, such as *Temora longicornis* consistently used a selective strategy, while diets of *Centropages hamatus* and *Acartia* spp*.* varied between stations, showing a trophic plasticity with the prey community. We found a dominance of Synechococcales reads in *Temora*’s gut content and a high prey diversity for the cladoceran *Evadne nordmanni*. Our study shows the wide range of prey species that supports mesozooplankton community and helps to understand the spatial and temporal complexity of plankton species interactions and discriminate the selectivity ability of four zooplankton key species. Due to the central role of plankton in marine waters, a better comprehension of the spatiotemporal variability in species interactions helps to estimate fluxes to benthic and pelagic predators.

## INTRODUCTION

From coasts to deep environments, marine ecosystems are composed of myriad species and the interplay among them structure food webs that transfer energy from primary producers to top predators (Azam *et al*., 1983; Pauly and Christensen, 1995; Stibor *et al*., 2004). Interactions among species in food webs have been one of the first processes studied in ecology (Elton, 1927). However, the understanding of the structure and dynamic, evaluation of species diet and trophic position is still challenging, particularly for planktonic food webs. This is largely due to the multitude of biotic interactions that may vary with environmental conditions and small size of the target organisms. In most biogeochemical models, zooplankton are represented in a single compartment (Fasham *et al*., 1990; Vichi *et al*., 2007), while pelagic food webs often use a trait-based approach, clustering species into size categories to describe trophic interactions (Woodward *et al*., 2005; Barnes *et al*., 2010). These approaches ignore the taxonomic and trophic variation among organisms within a size class. Zooplankton species have different shapes, size and feeding modes, causing variation in feeding inputs and trophic links. Some species of relatively similar size can occupy different trophic niches (Novotny *et al*., 2021), and as such the complexity of trophic interactions is often underestimated. In addition, many factors such as the feeding behavior (Jeschke *et al*., 2004) and the toxicity of the prey are influencing feeding interactions (Schultz and Kiørboe, 2009). There is a need for improved understanding of the planktonic trophic interactions in order to increase the precision of food web and biogeochemical models, as a change in planktonic community might affect carbon flow with consequences for global processes.

In the pelagic interaction network, zooplankton have a key role by concentrating and transferring carbon and essential biomolecules from primary producers to upper trophic levels and by shaping the phytoplanktonic community structure (Humes, 1994; Lampert and Sommer, 1997; Ohman and Hirche, 2001; Varpe *et al*., 2005; Winder *et al*., 2017). Zooplankton use various dietary resources of both autotrophic and heterotrophic origin (Kleppel, 1993). The gut content of zooplankton species often varies from prey composition in the surrounding water, suggesting a selective feeding behavior (Kiørboe *et al*., 1996; Selander *et al*., 2006). Mesozooplankton species occupying a similar size range use different feeding strategy, either a passive (i.e. ambush) or an active (i.e. feeding current or cruise feeding) feeding strategy (Tiselius and Jonsson, 1990; Kiørboe *et al*., 2010). Feeding strategies may differ depending on the prey availability, which is itself fluctuating with the environmental parameters and some species, such as *Centropages hamatus* and *Acartia* spp. can switch feeding behavior from passive to active (Tiselius and Jonsson, 1990; Kiørboe *et al*., 1996). These different feeding behaviors combined with various degrees of selectivity are inducing plasticity in trophic interactions in a mostly size-structured ecosystem (Kleppel, 1993; Sommer and Sommer, 2006). Zooplankton, that have different optimal environmental ranges, often experience varying prey composition at temporal and spatial scales, associated with seasons and changing environmental conditions, inducing variation in the network of trophic interactions at the base of the food web along spatial gradients.

**Fig. 1:**
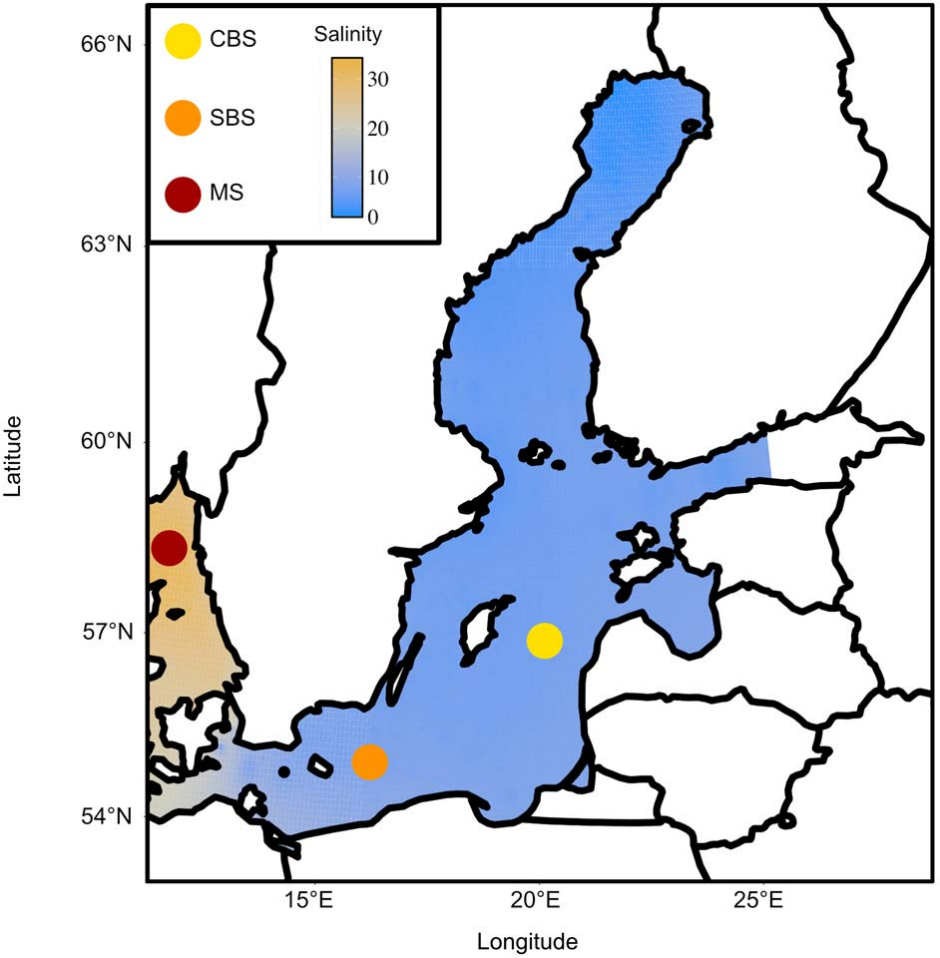
Location of the sampling stations, and sea surface salinity in the Baltic Sea and Skagerrak. Salinity surface data are retrieved from the Swedish national archive for oceanographic data (https://sharkweb.smhi.se/) with spatial interpolation using the function “idw” from the R package gstat (Pebesma and Wesseling, 1998).

The Baltic Sea is one of the largest brackish water bodies in the world with strong spatial and temporal variabilities caused by an increasing salinity gradient from north/east to south/west (Fig. 1) and strong seasonality. The mesozooplankton community comprises a diverse assemblage of mainly copepods and a few cladoceran species. Diatoms and dinoflagellates form the spring phytoplankton bloom when the water column starts to stratify and nutrients concentrations are high, while cyanobacteria dominate during the summer stratification period with low nitrogen availability. The Baltic Sea experienced one of the highest recorded temperature increases during the past century due to anthropogenic climate change (Bhend and von Storch, 2009; Rutgersson *et al*., 2014; Reusch *et al*., 2018). These modifications in abiotic parameters are altering the community structure and composition (Coutinho *et al*., 2019). For instance, an increase in the intensity of filamentous cyanobacteria summer bloom has been observed in the past decades (Huisman *et al*., 2018; Kahru *et al*., 1994), which potentially affects fluxes within the food web. The trophic role of cyanobacteria in the zooplankton diet and therefore in the Baltic Sea food web is still partially unknown. Due to the large size of cyanobacteria filaments, the ability of some species to produce toxins and/or their lack of essential nutrients, they are assumed to be an unpreferred prey item for many zooplankton grazers. However, several studies emphasize adaptations of zooplankton to coexist with cyanobacteria blooms (Ger *et al*., 2016). The influence of environmental conditions on the diet of mesozooplankton, despite their possible consequences on the whole food web, is understudied.

In this study, we analyze the dietary composition of four dominant primary consumers, including copepods and cladocerans, along the salinity gradient of the Baltic Sea using DNA metabarcoding. To disentangle the network of interactions, we used *16S rRNA* to identify phototrophic prokaryote and *18S rRNA* genes to identify photo-, mixo- and hetero-trophic eukaryote prey. We hypothesize that the different feeding behaviors will influence prey composition of the predators’ gut content and that the ability of zooplankton to select prey is varying across species. Moreover, we expect that copepods can switch feeding behavior depending on prey availability and that both picocyanobacteria and filamentous cyanobacteria, due to their high abundance, are included in the mesozooplankton diet and therefore, supporting the Baltic Sea pelagic food web.

## METHODS

### Sampling and species sorting

**Fig. 2:**
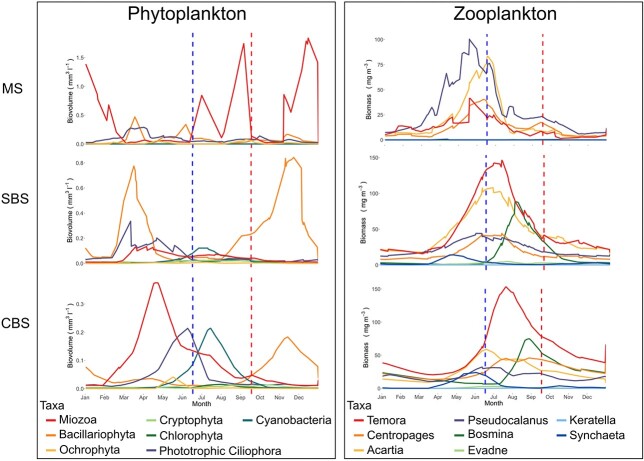
Seasonal dynamic of phytoplankton biovolume and zooplankton biomass in the different sampling stations based on daily average from 2006 to 2019. The data are available at the Swedish national archive for oceanographic data: https://sharkweb.smhi.se/. Samples are taken weekly to monthly during the spring and summer period and monthly during winter, and daily interpolated for average calculation. The blue and red vertical lines indicate the June and September sampling, respectively. The top panels represent the Marine station (MS), the middle represents the Southern Baltic Sea (SBS) and the lower the Central Baltic Sea (CBS).

Zooplankton and water samples were collected during two oceanographic cruises led by the Swedish Meteorological and Hydrological Institute (SMHI) that occur on a monthly basis. Three areas were sampled distributed along the north-east/south-west gradient of the Baltic proper and North Sea in June 2019, after the spring bloom for the marine station (MS) and the southern Baltic Sea station (SBS) and during the bloom for central Baltic Sea station (CBS), and in September 2020, after the summer bloom for the three stations (Figs 1 and 2). The monitoring stations BY16 and BY15 (here referred to as CBS) are located in the Gotland deep basin; stations BY5 and BY2 (referred to as SBS) are located in the Bornholm and the Arkona basins, respectively. The Å17 and Släggö stations are at high salinity in the North Sea (Fig. 1), referred to as MS. For DNA metabarcoding, two liters of integrated water samples from the surface to 20 m depth with three replicates were collected at each station, directly filtered using a peristaltic pump (Masterflex L/S) with 20, 2.0 and 0.2 μm pore size filters and subsequently frozen at −80°C until further analysis. Zooplankton were collected during day-time using vertical hauls with a 90 μm mesh size WP2 net, from 0 to 25 m (MS) and 0 to 30 m (CBS and SBS) depth, which represent the mixed surface layer at each station, and were immediately conserved in 95% ethanol at −20°C until further analysis. Individuals of the four most abundant mesozooplankton species *Acartia* spp*.* (including *Acartia bifilosa*, *Acartia longiremis* and *Acartia tonsa*), *Centropages hamatus*, *Temora longicornis* and *Evadne nordmanni* were sorted using a stereomicroscope (40X magnification) with five individuals per replicates and five replicates of each species at each station (Supplementary Table S1). Each individual was rinsed in three ethanol baths and one 1% bleach bath for 30 s in order to avoid DNA amplification of external symbionts (Zamora-Terol *et al*., 2020; Novotny *et al*., 2021).

### DNA extraction, PCR and sequencing

For the *16S* analysis, we amplified a 500 bp long fragment of the V3–V4 region of the *16S rRNA* gene (*16S*) using universal primers 341F-Adapter1 and 805R-Adapter2 targeting plastidial *16S* of phototrophic eukaryotes (Herlemann *et al*., 2011; Hu *et al*., 2016) as described in Novotny *et al*. (2021). DNA concentration and quality of post-PCR products was estimated using Qubit fluorometer (Qubit dsDNA BR Assay, Thermo Fisher) and a Bioanalyzer assay (Agilent, Santa Clara, California). Sequence clustering was done on MiSeq (MSC 2.5.0.5/RTA 1.18.54) pair-end setup (2 × 300 bp, version 3, Illumina, San Diego, California) with the addition of 10% genomic PhiX.

In order to reduce the number of copepod reads originating from the predator in the *18S* analysis, we performed a nested PCR, by adding an initial pre-amplification step the for zooplankton samples. For this purpose, we used a primer pair that amplifies an extended segment of the V4 region of the *18S* gene binding universally, but excludes calanoid copepods (non-copepod *18SF2:* AGCAGGCGGHAAATTRCCAATCY and non-copepod *18SR2:* CCGTGTTGAGTCAAATTAAGCCG) (Guo *et al*., 2012). In the pre-amplification step we used 10 μL of KAPA HiFi HotStart ReadyMix (Roche, KAPA Biosystem), 1 μL of each primer at a concentration of 10 μM, 6 μL of molecular grade water and 2 μL of extracted DNA per sample. The mix was processed through 2 min initial denaturation at 98°C followed by 5 PCR cycles with a denaturation of 20 s at 98°C, an annealing of 20 s at 68°C and an elongation period of 15 s at 72°C, followed by 10 cycles with a denaturation of 20 s at 98°C, an annealing of 20 s at 62°C and an elongation period of 15 s at 72°C. A final elongation was then performed at 72°C during one minute. The consecutive libraries for *18S* were constructed using the pre-amplified DNA as template according to best practice procedures described by Novotny *et al*. (2021).

Bioinformatic analyses were performed as described by Novotny *et al*. (2021), using cutadapt software (Martin, 2011) for primer removal and dada2 R package (Callahan *et al*., 2016) for quality control and filtering, error rate modeling, sequence dereplication, ribosomal sequence variant (RSV) inference and taxonomic assignment. The *16S* sequences were assigned to a custom-made database combining the SILVA *16S* reference database (Pruesse *et al*., 2007) with the PhytoREF database (Decelle *et al*., 2015) to achieve an adequate taxonomic resolution for both prokaryotes and photoautotrophic eukaryotes. For the assignation of the *18S* sequences we used the PR2 database (Guillou *et al*., 2013). Data can be found on European Nucleotide Archive website, under the project PRJEB52087.

### Data analysis

In this study, we used relative abundance of the DNA metabarcoding data to analyze the different gut content, which has been shown to represent diet data at population level (Deagle *et al*., 2019; Novotny *et al*., 2021). We applied a data quality filtering process on the metabarcoding data. For each combination of predator-station, the prey was selected if it was present in at least 40% of the replicates and if it represented at least 5% of the number of reads for all the replicates of one predator species at one station to exclude rare sequences. The *16S* data are shown at the class level. The *18S* data at the order level, except for the photoautotrophic species, which are presented at the class level.

### Data validation and visualization

Despite the nested PCR, the crustacean reads represented 72% of the total number of read for the *18S* analysis, which represent a major improvement in comparison to previous protocols (Zamora-Terol *et al*., 2020; Novotny *et al*., 2021) and increase considerably the reliability of the data. As the main goal of this study is to analyze the feeding interactions of the zooplankton prey, the crustacean reads were removed because it is more likely that it represents the predator itself rather than cannibalism, which we cannot differentiate with our method. For the *16S* data, heterotrophic bacteria, the epibiont Euglenozoa and the unidentified eukaryotic family were removed, and the unidentified Opisthokonta family and the parasitic Labyrinthulales within the *18S* data. We excluded these reads as we considered them as not being part of the predator diet (epibiont) or that the taxonomy was not precise enough for further analysis. Heterotrophic bacteria were excluded as more detailed analysis is needed to differentiate whether they are part of the prey or gut symbionts. We use *16S* and *18S* sequence data for comparison across taxonomic groups and stations. Quantification of diet overlap across stations and selectivity indices was based on *16S* only, as *18S* data are less quantitative given the range from single to multi-cell organisms (Novotny *et al*., 2021).

The statistical analysis and the visualization were conducted with R (R Core Team, 2020) using the R packages phyloseq (McMurdie and Holmes, 2013), associate with ggplot2 (Wickham, 2009) and circlize (Gu *et al*., 2014) for the circle plots. The circle plots are visualizing the diet of each predator across the different stations. The Adonis function from the package vegan (Oksanen *et al*., 2016) for the PERMANOVA and pairewiseAdonis (Martinez Arbizu, 2020) for the pairwise tests were used in order to compare the gut contents and the water samples. To analyze diet overlap between zooplankton species and highlight the variability of the diet across space and time, we used the Bray-Curtis distance based on the *16S* relative proportion of the mesozooplankton gut content. A low diet overlap across species shows a divergence of trophic niches while an elevated diet overlap for a similar species indicates an active selection of the food items.

### Selectivity index

To analyze the prey preference of the zooplankton, we used the selectivity index (Chesson, 1983) as suggested in Novotny (2021). The input data for this selectivity index are the *16S* sequence read abundance data both from the gut content and the water samples. The index is based on standardized forage ratio that can range between 0 and 1 with *Rij* representing the DNA sequence read abundance of the prey (*i*) in the gut content (*j*), *k* is the summation index, Rw*i* is the read abundance of the same prey in the water sample.


$$ {S}_{ij}=\frac{R_{ij}/{Rw}_i}{\sum_k{R}_{kj}/{Rw}_k} $$

## RESULTS

### Sequencing data and amplicon sequence variants

After the filtering process, we retrieved from the *16S* data, a total of 12 893 224 reads for 85 amplicon sequence variants (ASVs) in the gut content of the different zooplankton species and 213 ASVs in the water samples for 788 568 reads. For *18S*, we obtained a total of 2 345 403 reads with 133 ASVs in the gut content, and 3 444 488 reads with 1091 ASVs in the water samples. The mean number of *16S* reads per station was 48 242 for *Centropages*, 20 657 for *Acartia*, 40 019 for *Temora* and 4 817 for *Evadne*, and for the *18S* reads, 19 355 for *Centropages*, 62 254 for *Acartia*, 100 110 for *Temora* and 133 119 for *Evadne*. The reads represented the full prey spectrum from prokaryote to eukaryote, including photo- and hetero-trophic organisms.

### Water community

**Fig. 3:**
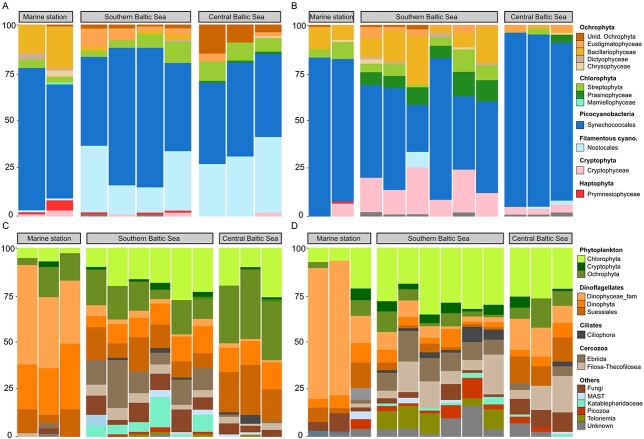
Relative abundance of sequence counts per phyla of the water samples, based on 16S rRNA (**A** and **B**) and 18S rRNA (**C** and **D**) gene reads. The left panels (A and C) represent data for June, the right panels (B and D) for September. Note that 16S data exclude reads from heterotrophic bacteria and 18S exclude crustacean reads. The bars represent individual biological replicates.

The water samples showed a relatively high similarity across stations according to the *16S* reads (Fig. 3A and B). The stations BY2 and BY5 were merged together as the water communities could not be differentiated (Permanova 2019: *P* = 0.068 and 2020: *P* = 0.186). The picocyanobacteria Synechococcales dominating the reads both in June and September, ranging from 44.6% to 86.3% of the total reads. In June, the filamentous cyanobacteria Nostocales were present in both the SBS and CBS with 27.0% and 37.2% of the reads, respectively, while Nostocales represented only 2.6% in the MS. Similarly, the Chlorophyta of the family Trebouxiophyceae occurred in all stations in June, while the proportion of Bacillariophyceae was decreasing with salinity, from 13.5% in the MS, 12.7% in the SBS and 3.3% in the CBS. In September, SBS had a high diversity, with the presence of Prasinophyceae (7.2%), Cryptophyceae (13.6%), Bacillariophyceae (12.7%) and Eustigmatophyceae (4.5%), but still Synechoccocales dominated with 48.2% of the reads (Fig. 3A and B).

According to the *18S* reads, the water community at the Baltic Sea stations (CBS, SBS) was dominated by both phototrophic species from the phyla Chlorophyta (from 18.8% to 29.1%) and Ochrophyta (from 4.9% to 32.9%) and the heterotrophs/mixotrophs from the family Dinophyceae (from 1.6% to 13.6%) and unclassified Dinophyta (from 4.7% to 13.3%) (Fig. 3C and D). The latter two taxa were dominating in the high-salinity stations (from 41.3% to 51.6% for the Dinophyceae and from 7.3 to 27.7% for the unclassified Dinophyta). The groups of microzooplankton Ebriida and Filosa-Thecofilosea represented 14.2% of the reads in the SBS in June and 13.5% in September, respectively (Fig. 3C and D), while Ciliophora were present mostly in SBS station with percentages up to 5.3% in September and in all three areas in June (up to 6.3%).

### Zooplankton diet patterns

**Fig. 4:**
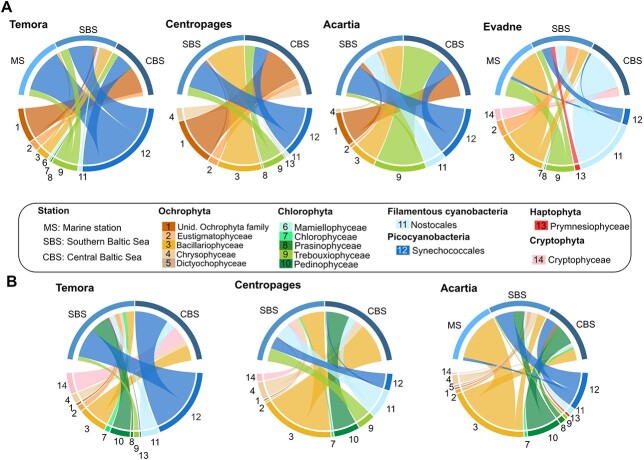
Feeding interactions per zooplankton species across the different stations based on the 16S rRNA gene reads from June (**A**) and September (**B**). The stations are represented on top of each circle plots and the preys on the lower part. The width of the segment represents the average relative read abundance associated to each predator.

The *16S* reads showed that all copepods were associated to Ochrophyta, Chlorophyta, Synechococcales and Nostocales, but in different proportions among stations and species. In June, the proportion of filamentous cyanobacteria (Nostocales) were neglectable for *Temora* and *Centropages*, but represented one quarter of the reads for *Acartia* in the SBS. Nostocales contribution increased gradually for *Evadne* from the most saline stations, where they were absent, to the SBS (26.1%) and the CBS (77.4%). In September, Nostocales reads were always lower than 10.1% for both *Temora* and *Acartia*, but contributed up to 24.2% for *Centropages* in the SBS (Fig. 4). The proportion of picocyanobacteria reads associated to *Temora* ranged from 30.9 to 74.3% (Fig. 4) and was lower for *Centropages* and *Acartia*, ranging from 2.2% to 37.0%, but remained higher than 16.5% for all the stations, except at CBS for *Centropages* and MS for *Acartia* in September. Unidentified members of the Ochrophyta family, represented an important proportion of the reads for both *Temora* (53.3%), *Centropages* (47.1%) and *Acartia* (21.3%) in June at CBS. The Bacillariophyceae family was identified in every zooplankton with relatively high proportion for *Centropages* (46.2%) and *Acartia* (28.1%) at SBS in June, and at MS for *Evadne* (61.4%). In September, they are the highest for *Temora* and *Centropages* at CBS (21.0% and 47.0%, respectively) and at MS for *Evadne* (92.4%). The Chlorophyta were mostly represented by Trebouxiophyceae in June, especially in CBS for *Acartia* (57.1%) and by Pedinophyceae in September (up to 39.7%).

**Fig. 5:**
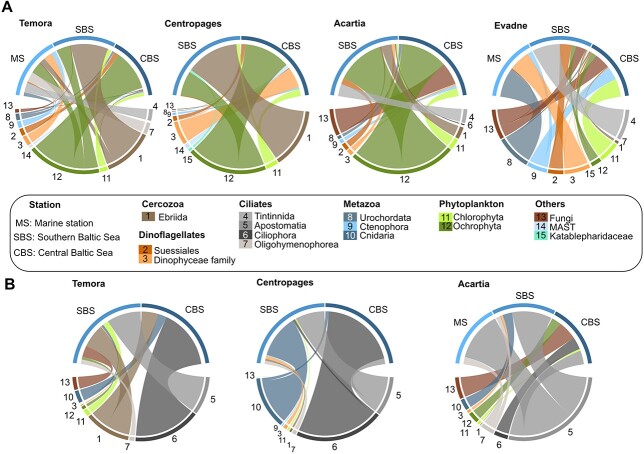
Feeding interactions of different zooplankton species across the different stations based on the 18S rRNA gene reads for June (**A**) and September (**B**). The stations are represented on top of each circle plots and the preys on the lower part. The width of the link represents the average relative read abundance associated to each predator.

For the *18S* data, we found a wide range of prey, including Chlorophyta, Ochrophyta and heterotrophic prey, such as ciliata, Cercozoa and some macroplanktonic organisms (metazoan) associated to all predators (Fig. 5). Dinoflagellates (Dinophyceae, Dinophyta and Suessiales) were mainly associated with *Centropages* and *Evadne* in June, with a maximum of 36.9% of the reads and had overall a low contribution with a mean of 5.5% of the reads across all species and the two sampling dates, despite its high frequency in the water samples ranging from 8.6% to 65.0% across stations and season.

In June, for the *18S* analysis, the proportion of phytoplanktonic prey varied between stations and between zooplankton species. The heterotrophic prey, such as the Cercozoa Ebriida, Urochordata, the ciliates order Tintinnida and Apostomatia, or Cnidaria dominated prey composition, with higher contributions in September than in June (Fig. 5). The summer blooming Cnidaria *Aurelia aurita* was identified as a prey item (up to 44.7%) across all stations and predators in September, while none of the predators had Cnidarian reads associated in June. Ciliates represented an important proportion of *Evadne*’s diet in June (up to 48.2%) and of all the predator species’ diet in September (up to 96.3%), although the proportion in the water was relatively low (up to 3.4%). The cladoceran *Evadne* has a variable diet across the salinity gradient with a high domination of the tunicate Appendicularia and dinoflagellates in the MS (48.0% and 37.0%, respectively), tintinnids ciliates in SBS (48.2%) and various prey in CBS with Ctenophora (16.8%), phytoplanktonic (47.1% including Chlorophyta and Ochrophyta) and fungi reads (27.2%). Each zooplankton species was associated with fungi (up to 33.8% depending on the station), which was strongest at CBS, except for *Centropages* in September, who has a slightly higher proportion at SBS then CBS (Fig. 5).

### Prey preference and diet overlap

**Fig. 6:**
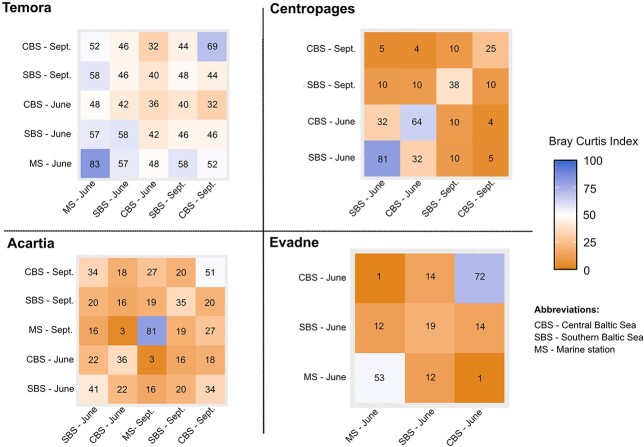
Bray Curtis similarity index (in percentage) of the gut content based on 16S analysis across stations and month for each zooplankton predator. A high value of the index represents a high similarity between the gut content. Each panel compares the diet of a mesozooplankton species at the different stations and cruises.

Gut content comparison within species using the Bray-Curtis distance showed that *Temora* had the most conservative diet across space and time, with 48% of similarity across all the samples, and the difference between station was not higher than within stations. In comparison, the mean values were 21% of similarity for *Centropages*, 25% for *Acartia* and 22% for *Evadne* (Fig. 6). Moreover, *Centropages* showed a low similarity between stations in September (10% similarity), but was higher in June (32%), while *Evadne* had a general low diet overlap across stations.

**Fig. 7:**
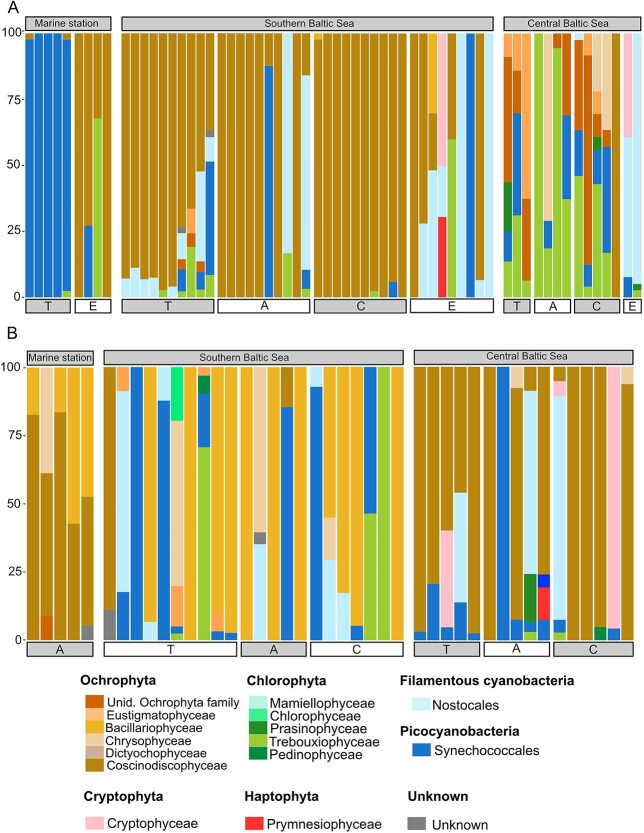
Preferred diet of the zooplankton predator based on the selectivity index calculated with 16S rRNA gene reads in the different stations for June (**A**) and September (**B**). Each bar represents a replicate of five organisms, the name of the predator is written under the bars: A for Acartia, T for Temora, C for Centropages and E for Evadne.

The selectivity index indicated the preferred prey species for the mesozooplankton predators (Fig. 7). At the MS, in June, *Temora* preferred almost exclusively Synechococcales (from 95.2% to 100%), while *Evadne* showed overall high preference for Coscinodiscophyceae (from 32.1% to 100%). At the same station in September, *Acartia* showed a preference for both Coscinodiscophyceae (from 47.2 to 83.5% depending on replicates) and Bacillariophyceae (up to 57.1%). At SBS, all copepod species preferred the diatoms in June and September (up to 100% for both), while *Evadne* showed a more diverse prey intake. At CBS, in June consumers preferred a broader range of prey, such as Eustigmatophyceae, Prasinophyceae, Chrysophyceae and Synechococcales, while the three copepod species selected Coscinodiscophyceae (up to 100%) at this station in September (Fig. 7).

## DISCUSSION

In this study, we developed an effective nested PCR protocol for the analysis of zooplankton gut content from selected organisms that avoids overrepresentation the consumers’ read abundance. This new tool provides more robust data to assess trophic interactions across diverse consumer species, which is an improvement from previous procedure (Ho *et al*., 2017; Zamora-Terol *et al*., 2020; Novotny *et al*., 2021). Moreover, the results elucidate the broad interactome of key components of the pelagic food web with high taxonomic resolution. To our knowledge, the combination of the analysis of gut content of mesozooplanktonic predators, using metabarcoding technics and an environmental gradient has not been conducted before. These data contribute to understand both the spatial variation of the diet at species level for *C. hamatus*, *E. nordmanni* and *T. longicornis* and at genus level for *Acartia*, the partitioning of the trophic niche among mesozooplankton species and the variation in the degree of selectivity of the different predator species.

### Differences in prey selectivity

Our results show that zooplankton prey composition was not proportional to prey availability in the water column, suggesting the ability for the predators to select prey. However, the degree of selectivity varied across species. We find a high diet overlap across all the stations for *Temora,* despite the variation in prey availability, suggesting diet stability and a more selective feeding behavior of this copepod. *Temora* had a high and stable input of picocyanobacteria, microzooplankton (cercozoan, ciliates) and diverse phytoplankton prey. This copepod species is described as an herbivorous filter feeder that can switch to carnivorous mode when the photoautotrophic community is low (Tiselius and Jonsson, 1990; Kleppel, 1993). This behavior is in agreement with our findings and high heterotrophic ciliate and ebriidan flagellate contributions were found in autumn, supporting that the feeding current behavior of *Temora* is efficient in catching heterotrophic prey (Jakobsen *et al*., 2005). Our data further support that *Temora* selects for diatoms when available (Dam and Lopes, 2003). Although diatom proportions were generally low in the water at our sampling events, *Temora* selected highly for Coscinodiscophyceae and Bacillariophyceae. This feeding adaptation likely enables *Temora* to be productive even if prey availability decreases, which is highlighted by their presence throughout the year.

In contrast, *Centropages*, *Acartia* and *Evadne* had a lower diet overlap between stations, which suggests an opportunistic non-selective feeding behavior. Particularly, *Centropages* diet varied with prey availability. *C. hamatus* is an omnivorous species able to switch feeding behavior from suspension feeding to ambush feeding (Hwang *et al*., 1994), with the feeding mode depending on the abundance of ciliates (Saage *et al*., 2009). This copepod species showed high selectivity for diatom prey, although their concentration was low in the water column. In September, *Centropages* preyed on a variety of heterotrophic prey, and in some stations almost exclusively on ciliates, suggesting a change of feeding strategy depending on the feeding environment.

The copepod *Acartia* and the cladoceran *Evadne* showed low values of diet overlap. *Acartia* is an omnivorous and suspension feeding copepod that can switch to ambush feeding at higher ciliate (or microzooplankton) abundance (Kiørboe *et al*., 1996) and can exert a high predation on ciliates (Jonson and Tiselius, 1990). This is supported in our study showing that prey composition was mainly comprised of autotrophic prey during June but ciliates contributed largely to prey composition in September. While copepod species are able to use “scanning current” (Kiørboe, 2011; Gonçalves *et al*., 2014) to select prey, the cladoceran *Evadne* is considered as a filter feeder (Katechakis *et al*., 2002). The opportunistic feeding behavior of *Evadne* is supported by a diverse diet composition, composed both of heterotrophic and autotrophic prey, agreeing with previous feeding studies (Katechakis and Stibor, 2004). This cladoceran species has short-term abundance peaks and was only observed in June with varying prey selectivity across stations, depending on availability.

Our results are supporting the study from Novotny *et al*. (2021) showing that *Temora* has a high diet overlap between samples on a seasonal scale, while *Acartia* and *Evadne* show similar diet and *Centropages* diet varies with availability. These data allow to identify the different trophic behavior of several key mesozooplankton species, from the specialist *Temora* to the generalist *Evadne*, with *Centropages* and *Acartia* in between. Overall, the feeding strategy of *Temora* appears to be effective in the Baltic Sea as this copepod species outcompetes *Acartia* and *Centropages* in the central and southern Baltic Sea by being the most abundant copepod in this region.

### Seasonal and spatial variation

While copepod feeding traits of ambush and filter feeders are suggested to vary consistently over seasonal and latitudinal scales (Kenitz *et al*., 2017; Prowe *et al*., 2018), our results show that coastal environments select for copepod species that can switch between feeding mode, depending on prey availability. Zooplankton species with active feeding strategies, such as filter feeding, are favored by non-motile phytoplankton prey, while dominance of heterotrophs favors passive feeding species, such as ambush feeders (Prowe *et al*., 2018). The *18S* data indicate that ambush feeding is preferred in September when the three copepod species *Temora*, *Centropages* and *Acartia* feed almost exclusively on heterotrophic prey, such as unicellular flagellates Ebriida and ciliates. In June, auto- and hetero-trophic prey contributed about equally to copepod diet. This suggests that the copepod species occupy a higher trophic position in September, feeding mainly on the microbial food web, inducing a lengthening of the trophic chain and therefore decrease food web efficiency. This highlights the essential position of copepods in both the microbial food web and classic food web (Kleppel *et al*., 1988; Sherr and Sherr, 1988) as well as the role of heterotrophic prey in sustaining food webs in coastal systems during time periods when phytoplankton abundance is low.

Besides variability of prey proportions among zooplankton species, our study shows variation at spatial scale. At the marine stations, picocyanobacteria, diatoms and ciliates contributed largely to the diet, while diverse phytoplankton prey are utilized at the Baltic Sea stations. The contribution of heterotrophic prey (Ebriida, Tintinnida, Cnidaria) was increasing with the salinity across the gradient (June: 38.4% (CBS) – 67.3% (SBS) – 90.0% (MS); September: 93.3% (CBS) – 96.3% (SBS) – 100% (MS)). In addition, zooplankton prey composition can be expected to vary across the vertical water column gradient. We focused on the water column above the thermocline where the majority of the selected zooplankton species are found. Investigating interactions across the vertical gradient is an important direction for expansion. Overall, our results emphasize the importance of microbial organisms in both brackish and marine waters since heterotrophic reads represented at least 38.4% of the total reads in the predator’s gut content, and illustrate the variation of feeding behavior together with environmental conditions.

### Cyanobacteria as prey for zooplankton

While the majority of the non-cyanobacteria prey was within the nano- and micro-plankton scale (data not shown), cyanobacteria prey size extents from picoplankton to filament-forming taxa. Picocyanobacteria reads of Synechococcales are found in all mesozooplankton gut content, with a relatively high proportion and strong selection of *Temora* at the most saline stations, and high intraspecific variability of Synechococcales selection for all other zooplankton species. Although direct grazing on picocyanobacteria is possible (Motwani and Gorokhova, 2013), they are not preyed upon effectively by copepods nor cladocerans (Frost, 1972; O’Connors *et al*., 1980). Synechococcales form colonies in the Baltic Sea (Callieri *et al*., 2011) and this uptake may be more efficient, particularly in June. However, high proportions of picocyanobacteria typically occur with high proportions of heterotroph reads, suggesting that most of the Synechococcales are taken up through secondary predation of heterotrophs, such as unicellular flagellates (Ebriida) and ciliates, although we cannot separate direct and indirect uptake with our approach. Nevertheless, the presence of picocyanobacteria in all mesozooplanktonic predators, with relatively high proportion for some species emphasizes the importance of the previously underestimated role of picocyanobacteria in supporting pelagic food webs.

Moreover, the *16S* data show that all copepod species were associated at least in one station with the filamentous cyanobacteria *Nostocales*, with increasing read proportions from *Temora* to *Centropages*, *Acartia* and *Evadne,* for the latter it is the main prey at CBS in June. These results suggest that *Temora* is able to select and actively avoid the filamentous cyanobacteria (Gonçalves *et al*., 2014) and that this capability is present, but not as efficient for *Centropages* and *Acartia*, and absent for *Evadne*. Although filamentous cyanobacteria are often described as being unpalatable for zooplankton, more recent studies support our findings showing significant cyanobacteria ingestion by diverse zooplankton species (Ger *et al*., 2016; Zamora-Terol *et al*., 2020; Novotny *et al*., 2021). The uptake of cyanobacteria is further supported by biomarker studies, showing that cyanobacteria-derived macromolecules are incorporated in mesozooplankton predators (Eglite *et al*., 2019).

### Degraded organic matter

We find high associated read numbers of the copepod species with the cnidarian *A. aurita* at all stations in September. Given its large size (10–25 cm), we assume feeding on egg and larvae stages that are within the size of 120 μm (Goldstein and Riisgård, 2016) or on degraded cnidarian material (Steinberg *et al*., 2008). Both of these explanations could explain the higher proportion in September than in June, due to the jellyfish life cycle and population decline after the summer period (Schulz *et al*., 2012). While the role of degraded matter in the food web is not fully understood (Jackson and Kiørboe, 2004; Turner, 2014), zooplankton grazing on degraded material can be considerable (Schnetzer and Steinberg, 2002; Hernández-Leòn *et al*., 2020) and is likely an important diet contribution in the Baltic Sea (Koski *et al*., 2005). The presence of cnidarian reads shows the importance of the jellyfish input to the copepods diet after the summer bloom period, when other sources of food are scarce.

The *18S* data also show that, at the Baltic Sea stations, *Acartia*, *Temora* and *Evadne* were associated with fungi. These could be taken up indirectly with fungi-associated prey items or by ingestion of degraded material (Jobard *et al*., 2010). The presence of fungi in zooplankton diets suggests that fungi are abundant in the pelagic food web, both in June and September. However, their function and role in zooplankton diets needs further investigations (Sime-Ngando, 2012). These findings suggest that zooplankton contribute to the recycling of organic matter within the pelagic environment and transport of degraded material to higher trophic levels. Moreover, the diverse prey assemblages found associated to copepods highlight their adaptability to live all year round in a highly seasonal environment.

## CONCLUSION

This study shows the wide prey range of mesozooplankton species and that different predator species of the same size range are responding differently to varying prey composition. *Temora* mostly keeps its selective behavior, while the other zooplankton species feed opportunistically on diverse prey. However, all the copepod species are increasing their input of heterotrophic prey in autumn, when phytoplankton production is low. Moreover, we show that both picocyanobacteria and filamentous cyanobacteria are supporting the Baltic Sea food web, Synechococcales for the more selective organisms and Nostocales are a prey of the less selective consumers. Finally, we illustrate the diversity of feeding behavior and consequently trophic niches among mesozooplankton and show that trait-based models are useful but more information about trophic interactions, and their variations with prey availability, are needed to understand major concept, such as the efficiency of trophic transfer and the biological carbon pump.

## Supplementary Material

Supplementary-Table_S1_fbad007

## Data Availability

Data can be found on European Nucleotide Archive website, under the project PRJEB52087.
