# Correction to: Opportunistic vs selective feeding strategies of zooplankton under changing environmental conditions

**DOI:** 10.1093/plankt/fbae009

**Published:** 2024-02-27

This is a correction to: Baptiste Serandour, Kinlan M G Jan, Andreas Novotny, Monika Winder, Opportunistic vs selective feeding strategies of zooplankton under changing environmental conditions, *Journal of Plankton Research*, Volume 45, Issue 2, March/April 2023, Pages 389–403, https://doi.org/10.1093/plankt/fbad007

The originally published version of this manuscript required correction.

In the Methods section, under subheading “DNA extraction, PCR and sequencing”, the authors wished to correct the following text, in order to give credit to a previously published primer set they used:

For this purpose, we designed a novel primer pair (non-copepod *18SF2*: AGCAGGCGGHAAATTRCCAATCY and non-copepod *18SR2*: CCGTGTTGAGTCAAATTAAGCCG) that amplifies an extended segment of the V4 region of the *18S* gene binding universally, but excludes calanoid copepods. The primers were designed using Prime3 software and tested for specificity against the Protist Ribosomal Reference (PR2) database (Guillou et al., 2013).

This text has been corrected as follows:

For this purpose, we used a primer pair that amplifies an extended segment of the V4 region of the 18S gene binding universally, but excludes calanoid copepods (non-copepod 18SF2: AGCAGGCGGHAAATTRCCAATCY and non-copepod 18SR2: CCGTGTTGAGTCAAATTAAGCCG) (Guo et al., 2012).

The citation included in the above sentence has been added to the manuscript's reference list as follows:

Guo, Z., Liu, S., Hu, S., Li, T., Huang, Y., Liu, G., and Lin, S. (2012) Prevalent ciliate symbiosis on copepods: High genetic diversity and wide distribution detected using small subunit ribosomal RNA gene. PLoS ONE 7(9): e44847. https://doi.org/10.1371/journal.pone.0044847

