# Supplementary material for: Opportunistic vs selective feeding strategies of zooplankton under changing environmental conditions
Source: J Plankton Res. 2023 Feb 22;45(2):389–403. doi: 10.1093/plankt/fbad007 (PMC10066809; doi:10.1093/plankt/fbad007)
Supplement: Supplementary-Table_S1_fbad007 [file supplementary-table_s1_fbad007.docx]

|  | | Predator species | Marine station | | Southern Baltic Sea | | Central Baltic Sea | |
| --- | --- | --- | --- | --- | --- | --- | --- | --- |
|  |  |  | June | September | June | September | June | September |
| 16S | Replicates sampled | Acartia | 0 | 5 | 10 | 5 | 5 | 5 |
|  |  | Centropages | 0 | 0 | 10 | 10 | 5 | 5 |
|  |  | Evadne | 5 | 0 | 10 | 0 | 5 | 0 |
|  |  | Temora | 5 | 0 | 10 | 10 | 5 | 5 |
|  | Replicates after filtering process | Acartia | 0 | 5 | 10 | 5 | 4 | 5 |
|  |  | Centropages | 0 | 0 | 10 | 7 | 5 | 5 |
|  |  | Evadne | 4 | 0 | 8 | 0 | 2 | 0 |
|  |  | Temora | 5 | 0 | 10 | 10 | 3 | 5 |
| 18S | Replicates sampled | Acartia | 0 | 5 | 10 | 10 | 5 | 5 |
|  |  | Centropages | 0 | 0 | 10 | 10 | 5 | 5 |
|  |  | Evadne | 5 | 0 | 10 | 0 | 5 | 0 |
|  |  | Temora | 5 | 0 | 10 | 10 | 5 | 5 |
|  | Replicates after filtering process | Acartia | 0 | 2 | 10 | 3 | 5 | 2 |
|  |  | Centropages | 0 | 0 | 10 | 6 | 5 | 2 |
|  |  | Evadne | 4 | 0 | 10 | 0 | 5 | 0 |
|  |  | Temora | 5 | 0 | 10 | 4 | 5 | 3 |
| Abiotic parameters | Temperature (°C) | | 11.42 | 16.99 | 17.31 | 13.45 | 7.07 | 6.02 |
|  | Salinity | | 25.65 | 26.24 | 9.78 | 10.39 | 5.33 | 7.15 |
|  | Latitude | | 58.28433 | | 55.25002 | | 57.31233 | |
|  | Longitude | | 10.50433 | | 15.98327 | | 20.07583 | |

*Table S1: Number of replicates before and after filtering process. Each replicate represents a group of 5 predators pooled together. The lower part of the table shows the temperature, salinity and the localisation of the sampling stations.*
